# Opportunities to improve environmental sustainability of pork production through genetics

**DOI:** 10.1093/jas/skaf042

**Published:** 2025-05-27

**Authors:** Jack C M Dekkers

**Affiliations:** Department of Animal Science, College of Agriculture and Life Sciences, Iowa State University, Ames, IA 50011-3150, USA

**Keywords:** environmental sustainability, genetics, genetic improvement, pork ecosystem

## Abstract

Breeding programs in pigs primarily aim to reduce the cost of production but, because of the strong parallel effects of traits on the cost of production and environmental impacts, current breeding programs also substantially reduce the environmental impacts of pork production, although these reductions may be overestimated if the possible use of manure as a source of fertilizer is not accounted for. The purpose of this review is to summarize and explore opportunities that exist to further enhance these impacts by 1) changing the emphasis on traits in breeding programs, 2) including new traits, 3) integrating genetics and nutrition, and 4) transgenics and gene editing. Apart from accelerating rates of gain for productivity and efficiency at the commercial level, limited opportunities exist to further enhance reductions in environmental impacts by changing the emphasis on traits that are currently under selection, because of the high correlation between the impacts of these traits on cost of production and environmental impacts. However, opportunities exist to include traits related to resilience to disease and heat stress, methane emission (enteric and manure), and the efficient use of dietary nitrogen (N), phosphorus (P), and other ingredients, which all appear to have a genetic component. The limited research available to date suggests that genetic differences in efficiency and environmental impacts are smaller when pigs are fed diets that are tailored to their nutritional requirements, suggesting fewer genotype by diet interactions under such nutritional strategies. Selection for performance when fed diets that are tailored to meet the specific nutritional requirements of the line, or even the individual pig, can direct selection pressure to improvement of efficiency of the use of specific diet components. The effectiveness of this approach, however, depends on the accuracy of the nutritional models that are employed to determine nutrient requirements, as well as the accuracy with which these requirements can be characterized at the genetic level. Opportunities also exist to use transgenics or gene editing to provide solutions for anti-nutritional factors that many feedstuffs contain. Most emphasis on further reducing the environmental impact of pork production through genetics should focus on the grow-finish phase because it has the largest environmental impact and mitigation opportunities. Although this is expected to translate into additional reductions in environmental impacts of the reproduction phase, research into genetic selection or interventions that are specific to this phase is also needed.

## Teaser

By improving productivity and efficiency, genetics has made substantial contributions over the past decades to reducing the carbon footprint of pork production. This review explores opportunities to further enhance the impacts of genetics on sustainability by including new traits in breeding programs, by integrating genetics and nutrition, and through the use of gene editing.

## Introduction

Climate change, combined with the large contribution of livestock production to greenhouse gas (**GHG**) emissions and other environmental impacts, requires a concerted effort to mitigate these impacts. This includes animal nutrition, animal and manure management, and animal breeding.

Animal breeding has been highly successful in improving the productivity and efficiency of the modern pig, both in terms of growth performance, as well as reproductive performance (Neeteson et al. 2013). This has substantially reduced the cost of production (**CoP**). Modern breeding programs use a multi-trait selection approach, balancing genetic progress in growth rate, feed efficiency, and meat quality of the grow-finish pig, and litters per sow per year, litter size, and prewean mortality and growth rate on the maternal side (Neeteson et al. 2013; Knap et al., 2023). Modern breeding programs achieve this balance by recording the relevant phenotypes, estimating breeding values (**EBV**) on purebred selection candidates, nowadays aided by genomics, and by assigning relative weights to each trait to construct a selection index as a weighted average of the EBV for each trait, which is used as the main selection criterion. The success of this balanced breeding approach is well-documented (Knap et al., 2023).

In current breeding programs, weights assigned to each trait are primarily determined by economics, aiming to optimize profit per kilogram of pork or minimize the CoP per kilogram of pork (Amer et al., 2014; Hermesch et al., 2014). These so-called economic values are derived as the increase in profit or CoP when increasing the mean of the trait by one unit (or one genetic standard deviation), keeping the mean of all other traits considered constant. It is well-known that increasing productivity and feed efficiency results in substantial reductions in the environmental impact of livestock production by requiring less feed to produce a unit of product, primarily through dilution of maintenance requirements across a larger amount of product per animal. Gjerlaug Enger et al. (2022) estimated the annual reduction of GHG from Norwegian pork production as a result of genetic improvement to be 1.4% of the mean of 186 kg CO_2_ eq for a pig with a carcass weight of 80 kg for the maternal Landrace breed and 1.9% for the terminal Duroc breed. Most of these reductions were the result of genetic improvement in finisher feed efficiency, in particular for the Duroc breed (approximately 1.6% and 0.7% for Duroc and Landrace, respectively), followed by the number of piglets weaned/sow/yr for the Landrace breed (approximately 0.5%), and finishing mortality (0.2% and 0.1% for Duroc and Landrace, respectively). The large contribution of feed efficiency reflects the fact that 80% of GHG from Norwegian pork production originates from feed production, coinciding with feed representing approximately 75% of variable costs of pork production (Gjerlaug Enger et al., 2022). In 2008, the reduction in global warming potential (**GWP**) by pork production in the United Kingdom over the previous 34 yr as a result of genetic improvement was estimated at 26%, i.e., about 0.8%/yr (DEFRA, 2008). A recent life cycle assessment (**LCA**) analysis based on expected rates of genetic improvement for one breeding company (Thoma et al., 2024) predicted reductions in GWP and other environmental impact categories of 0.8 to 1%/yr over the next 9 yr, i.e., similar to historical estimates. Given the largely similar breeding goals across countries and breeding programs, similar trends are expected in other countries and across genetic programs. Thus, current breeding programs, which are primarily aimed at reducing CoP, have and continue to contribute substantially to reducing the environmental impacts of pork production. Against this background, the purpose here is to review additional opportunities that exist to further enhance the environmental sustainability of pork production through genetics by 1) changing the emphasis on traits in current programs, 2) including new traits in phenotype recording and selection programs, 3) integrating genetics and nutrition, and 4) genetic modification or gene editing.

## Opportunities to enhance sustainability by changing the emphasis on traits in current breeding programs

Some studies have attempted to incorporate the impact of genetic improvement on GHG production into breeding programs by assigning a value to GHG production based on shadow price calculations (Ali et al., 2018; Alfonso, 2019), as suggested by Amer et al. (2018), and with the shadow price of CO_2_ equivalents computed following Ali et al. (2017). The study by Ali et al. (2018), which modeled pork production in Brazil, considered traits related to reproductive efficiency (number born alive, preweaning mortality, and wean-oestrus interval) and traits related to growth efficiency (average daily gain, **ADG**, and feed conversion ratio, **FCR**) for the breeding program. They found that incorporating the cost of GHG emissions into economic value calculations increased the (absolute value) of the economic values of growth efficiency but reduced the economic importance of improving reproductive efficiency, when expressed on a farm basis with a fixed number of sows and a fixed number of pigs sent to market at a fixed slaughter age (see their Table 2). The reason for the reduced importance of reproductive performance was that more productive sows and their piglets need more feed and produce more manure, while reducing days to market weight and improving grow-finish FCR reduces feed required and manure produced per farm. However, when economic values were expressed on a per finished pig basis (or, equivalently, on a per kilogram of pork basis), which is the relevant scale, incorporating the cost of GHG production also increased the economic value of traits associated with reproductive efficiency because improvements in reproductive performance of sows allow GHG emissions associated with the sow farm to be spread over a larger number of finished pigs. However, economic values for reproduction traits still increased proportionally less (by 4.5% to 6%) than those for growth efficiency traits (8.0% for ADG and 10.9% for FCR). As a result, the proportional emphasis on reproduction vs. growth efficiency traits (i.e., the relative economic values) dropped slightly, from 28.3% vs. 71.7% to 27.4% vs. 72.6% (Ali et al., 2018).

A similar conclusion was reached by Alfonso (2019), who considered only sow reproduction traits but with a more comprehensive set of traits. Similar to Ali et al. (2018), GHG emissions were incorporated into economic values through the shadow price of GHG. Results indicated that inclusion of GHG emissions diminished the relative economic values of litter size and piglet survival vs. traits that reduced the number of nonproductive days, i.e., age at first conception and interval from weaning to conception. The reduction in relative economic values for litter size and piglet survival can be explained by the impact of increasing the number of piglets per litter on feed required and manure produced. However, despite an increase in GHG emissions per litter, improving these traits still reduced GHG emissions per piglet weaned and, therefore, per kilogram of pork, because emissions associated with a litter are spread over a larger number of piglets weaned.

Many studies on multi-trait breeding programs have shown that responses in individual traits from selection on an index tend to be robust to changes in relative economic values, especially if traits under selection are genetically correlated. This was evaluated by Ali et al. (2019) by predicting responses to selection in the terminal sire and maternal lines using economic values that did or did not incorporate the shadow price of GHG for a typical farrow-to-finish farm in Brazil. Results showed that, indeed, responses to selection in individual traits were little affected by incorporating the cost of GHG emissions, with selection responses using both sets of relative economic values resulting in similar reductions in GHG emissions and N and P excretions per finished pig. Specifically, one round of selection reduced GHG emission by 1.13 kg CO_2_ equivalents per slaughter pig, or 0.5% per generation (following calculations by Knap, 2022). Results also showed, however, that selection in terminal sire lines, for grow-finish traits resulted in much greater reductions in GHG emissions and N and P excretions than selection in maternal lines, which is for a combination of reproduction and grow-finish traits. Thus, genetic improvement to reduce the environmental impact of pork production is more important for terminal sire line programs. A similar conclusion was reached by Gjerlaug Enger et al. (2022) for pork production in Norway, as described in the introduction. It should be noted, however, that these studies did not consider several important traits in maternal line programs that are associated with gilt rearing, including age at first farrowing and sow longevity (i.e., replacement rate), which have a direct impact on feed requirements and manure production per finisher pig.

**Figure 1.**
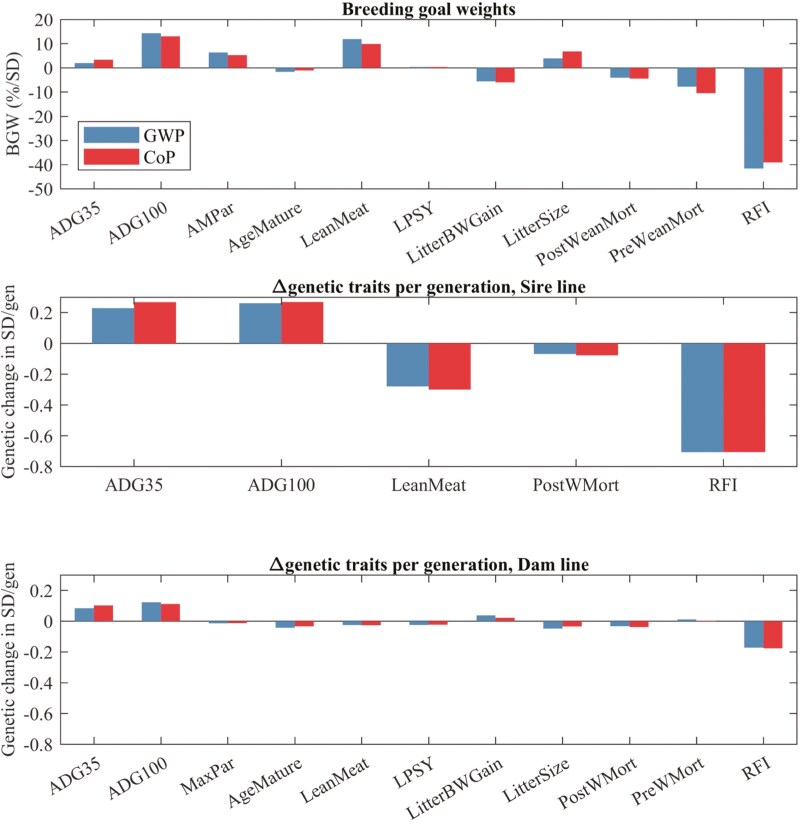
(based on Fig. 4.7 of Ottosen, 2021) Standardized breeding goal weights evaluated at baseline trait means (A) and expected genetic change in traits per generation for the sire line (B) and for the dam lines (C). The breeding goals are to reduce GWP or Cost of production (CoP). Expected genetic gains in the crossbreds (not shown) are the average of expected genetic gains in the sire and dam lines. Standardized breeding goal weights are expressed as a % of the sum of the absolute values of breeding goal weights. Genetic change per generation is expressed in units of genetic standard deviations (SD). Traits included in the breeding goal are ADG35, average daily growth rate from weaning to start of the grower phase around 35 kg; ADG100, average daily gain in the finisher phase, MaxPar, number of parities per sow; AgeMature, age at first insemination; LeanMeat, lean meat %; LPSY, litters per sow per year; RFI, residual feed intake; LitterBWGain, body weight gain of the litter from birth to weaning; LitterSize, number born alive per litter; PreWMort, mortality rate for live born piglets from birth to weaning; PostWMort, the mortality rate for pigs from weaning until slaughter.

Rather than incorporating environmental aspects in ongoing breeding programs by including the costs of environmental impacts associated with traits based on the shadow price of CO_2_ equivalents, added to the impact of each trait on profit, as in Ali et al. (2018, 2019) and Alfonso (2019), Ottosen et al. (2020a) used an LCA to evaluate the direct impact of genetic change in individual traits on multiple environmental impact categories, including GWP, terrestrial acidification potential (**TAP**), freshwater eutrophication potential (**FEP**), agricultural land use (**ALU**), and fossil resource scarcity (**FRS**). They also included the impact of genetic correlations among traits on these impacts, mimicking the impact of single-trait selection for each trait. However, for the purpose of multi-trait genetic improvement, the weight that must be assigned to a trait in the breeding goal must reflect the impact on the breeding objective (e.g., profit or GWP) of increasing the population mean for that trait, while keeping the mean for all other traits in the breeding goal constant (Hazel, 1943). I.e., weights on traits in the breeding goal should reflect partial regression coefficients of trait means on the breeding objective. In standard index selection theory (Hazel, 1943), genetic correlations among traits are used when deriving responses to selection on the resulting multi-trait index, rather than for deriving the weights in the breeding goal. Such a selection index approach of deriving weights and responses to selection was applied by Ottosen et al. (2020b) and Ottosen (2021) for different breeding objectives, including minimizing GWP and minimizing the CoP, with the latter representing the main goal of current breeding programs. Compared to previous studies, Ottosen et al. (2020a,b) and Ottosen (2021) also considered a more comprehensive set of traits, including the reproduction traits of age at sexual maturity, litters per sow per year, longevity, litter size, litter gain, and prewean mortality, and the grow-finish traits of ADG in the nursery and finisher, lean meat %, postwean mortality, and residual feed intake (**RFI**), a measure of feed efficiency computed as the difference between observed and predicted feed intake based on weight, growth rate, and backfat, with lower values corresponding to better feed efficiency (Koch et al., 1963; Cai et al., 2008). Average daily feed intake (**ADFI**) and backfat thickness were included in the selection index but not in the breeding goal and were, thus, assumed to not directly affect CoP and environmental impacts beyond the other traits that were in the breeding goal, including ADG, lean meat %, and RFI. In addition, rather than using standard diets, least-cost rations that met energy and protein requirements were developed for each production phase and in each generation (subject to genetic change), using the prevailing conditions at the time in Denmark or the United Kingdom. Relative weights assigned to traits when the breeding objective was to minimize GWP were compared to those derived based on the objective to minimize CoP. The top graph in Fig. 1 (Fig. 4.7 from Ottosen, 2021) shows a comparison of the resulting sets of relative weights. Differences were limited, similar to the impact of incorporating a shadow price for CO_2_, as pursued by Ali et al. (2018) and Alfonso (2019); the correlation between the 2 sets of relative weights was 0.99. Also, similar to Ali et al. (2018), relative weights were slightly larger (in absolute terms) for grow-finish traits (ADG, lean meat %, and RFI), and slightly lower for sow reproduction traits (litter size, litter gain, prewean mortality) when the breeding objective was GWP compared to the CoP objective. Also similar to results by Alfonso (2019), the use of GWP as the breeding objective increased the relative weights (in absolute value) for traits that were related to nonproductive days (i.e., age at first estrus and sow longevity, in terms of number of parities).

The bottom 2 graphs of Fig. 1 show expected responses in the first generation on the 2 alternate sets of relative weights in the terminal sire line and in the maternal dam line. Generally, responses were very similar between the 2 sets of relative weights for both lines. As expected, responses for grow-finish traits were considerably larger for the sire line than for the dam line because all emphasis is on grow-finish traits in the terminal sire line. Note that expected responses in the crossbred grow-finish pigs are the average of the responses in the sire and dam lines, assuming unit genetic correlations between performance in purebreds and crossbreds.

**Figure 2.**
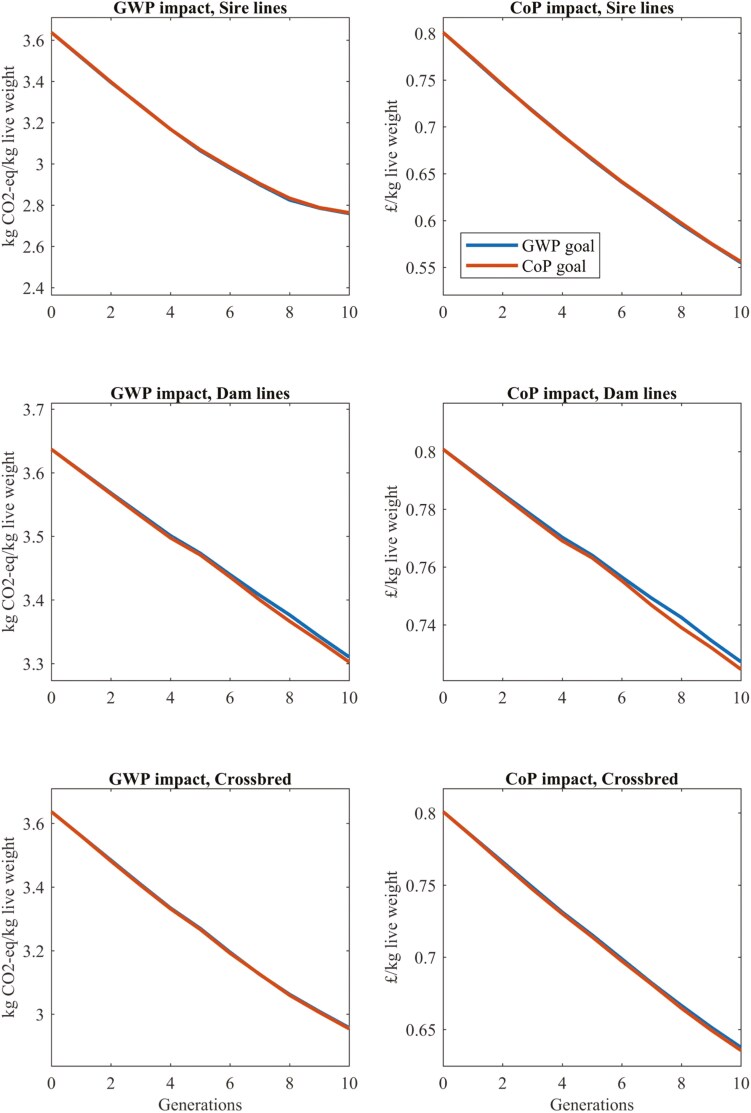
(based on Fig. 4.10 of Ottosen, 2021) The effect of 10 generations of selection in the sire line and in the dam lines for either GWP or Cost of Production (CoP) on outcomes per kilogram of live weight at the farm gate for purebreds and crossbreds.

Fig. 2 (Fig. 4.10 of Ottosen, 2021) shows the effect of 10 generations of selection on either set of relative weights on GWP and CoP per kilogram of live animal at farm gate in the sire and dam lines and the crossbreds. Differences in responses between the 2 sets of relative weights were minimal, as expected based on trait responses. Results show that 10 generations of selection on either set of relative weights reduced CoP at the crossbred level by 20.5% and GWP by 18.7%/kg live weight at farm gate, with approximately 75% of both of these responses resulting from selection in the sire line. Using parameters relevant to the UK, predicted reductions in GWP were slightly smaller, at 15.1% over 10 generations.

Reductions in GWP from genetic improvement predicted by Ottosen et al. (2020b) and Ottosen (2021) were more than 3 times greater than the 0.5% reduction per generation derived by Knap (2022) based on results by Ali et al. (2018) from incorporating the shadow price for CO_2_, but similar to estimates obtained by Gjerlaug Engler et al. (2022) for pork production in Norway. Based on a review of literature, MacLeod et al. (2019) estimated that the emission intensity of pig meat in the United Kingdom could be reduced by around 0.3%/yr if ‘rebound effects’, such as an increase in sow replacement and mortality rates, could be avoided. Using simulation for conditions in Sweden, Zira et al. (2022) showed that a breeding goal that included considerations of sustainability, based on Ottosen et al. (2020a), among others, and animal welfare, based on Wallenbeck et al. (2016), resulted in a reduction of GHG per kilogram of pork by 0.89%/yr, compared to 0.62%/yr for the current breeding goal.

It should be noted that most LCA, including those used by Ottosen et al. (2021) and Ottosen (2021), ignore the possibility that manure can be a source of fertilizer and, thereby, offset some of the cost and impact of synthetic fertilizers used in feed production. Taking this into account will reduce the estimates of the positive environmental impacts of genetic improvement for productivity and efficiency.

The reason for the greater impact of genetic selection on reducing GWP predicted by Ottosen et al. (2021) compared to other literature may be the inclusion of additional traits associated with efficiency, as well as their optimization and re-optimization of diets across generations. With regard to the latter, based on several lines of evidence, Knap (2022) argued that “more advanced genotypes require more advanced feeding strategies to bring their more sustainable performance potential to expression.” This may also explain the greater impact of genetic improvement on GWP predicted for Denmark than for the United Kingdom by Ottosen (2021), although absolute impacts of pork production on GWP were greater for Denmark than for the UK, both before and after 10 generations of selection. The higher production levels in Denmark compared to the United Kingdom result in greater requirements for nutrient-dense feeds, which, combined with the availability of different feed ingredients in Denmark, resulted in a greater reduction in GWP from genetic improvement in Denmark, both relative and in actual numbers.

Feed intake capacity is an important determinant of the impact of pork production on GWP. In this regard, an important assumption by Ottosen et al. (2020b) and Ottosen (2021) was a constant energy concentration in the feed for grow-finish pigs and that pigs adapt their feed intake entirely according to the nutrient density of the feed. Thus, feed intake was modeled based on average energy requirements for growth and maintenance, in combination with RFI. As a result, when the selection on a trait reduced the energy requirements for a production phase, feed intake decreased in proportionate amounts. Feed intake depends on many factors and can therefore be challenging to predict. Although pigs are expected to regulate their feed intake relative to the first limiting nutrient resource content in the feed, this is not true in all cases (Li and Patience, 2017). To evaluate the impact of the feed intake assumptions, Ottosen (2021) investigated 3 alternative feed intake scenarios: I) feed intake was held constant at the 2017 commercial average value; II) feed intake was adjusted according to the predicted ADFI trait in the breeding simulation; and III) feed intake was adjusted exactly for the first limiting nutrient in the feed, so both feed composition and feed intake were optimized for least cost (i.e., precision feeding). The alternative feed intake assumption scenarios affected environmental impacts but, again, differences between breeding objectives were minor for scenarios I and III because both resulted in minor changes in ingredient inclusions. However, for scenario II, substantial differences between breeding objectives were observed because of large changes in feed ingredient contents, with the GWP breeding objective resulting in a substantially greater reduction in GWP than the CoP breeding objective. This emphasizes the need for additional research into the genetic control of feed intake and its interactions with diet formulation.

Ottosen et al. (2020b) and Ottosen (2021) also investigated the impact of setting other environmental impact categories as the breeding objective, including TAP, FEP, ALU, and FRS. Although some environmental impact categories were found to be reduced more than others, the difference in responses to selection between the alternate breeding objectives was relatively minor, similar to differences observed for GWP and CoP as breeding objectives. The main reason for these minor differences was the small differences in the impacts of the feed ingredients used, noting that least-cost was the criterion used to formulate the diet for all breeding goals, which, under current price scenarios, tended to favor the inclusion of high nutrient density ingredients rather than alternative feed ingredients such as insect meal, algal meal, yeast protein concentrate, etc. Changes in ingredient prices, or formulating diets considering environmental impacts, as was done by Garcia-Launay et al. (2018), will affect the impact the effect of selection (especially for feed efficiency) on CoP and on environmental outcomes but may not affect the minor differences between breeding objectives that were observed by Ottosen et al. (2020b) and Ottosen (2021).

This section of the review demonstrates that current breeding programs already substantially contribute to reducing the environmental impact of pork production and that opportunities to further accelerate these reductions based on traits that are currently routinely recorded in breeding programs, i.e., ADG, feed efficiency, backfat, reproductive performance, and mortality under standard conditions, are limited. This excludes opportunities to further accelerate rates of genetic gain, especially for feed efficiency and mortality, by incorporating genomic selection and other technologies that can enhance breeding programs, such as enhanced reproductive technologies, and by obtaining more heritable phenotypes for key traits in the breeding goal.

## Opportunities to enhance sustainability by improving phenotype recording for current breeding goal traits.

### Phenotypes on crossbreds in commercial environments

Traditionally, most pig breeding programs have relied on phenotypes recorded on purebred animals in high-health nucleus herds to inform selection decisions. However, substantial research has shown that the genetic correlation between a trait (e.g., ADG) recorded on crossbreds in commercial herds and that same trait recorded on purebreds in a nucleus environment can be substantially less than one (Wientjes and Calus, 2017). Average estimates of genetic correlations by trait category were 0.54 for reproduction traits and ranged from 0.66 to 0.69 for other traits, including feed and growth traits (Wientjes and Calus, 2017). These genetic correlations quantify the portions of the genetic improvements that are achieved at the purebred nucleus level based on selection on purebred performance in the nucleus that are expected to be expressed at the commercial level. Thus, an additional opportunity to enhance rates of genetic improvement in commercial pork production systems for current breeding goals is to collect phenotypes on crossbreds in commercial environments and use these phenotypes to compute EBV for purebreds in the nucleus. Such strategies are greatly facilitated by using genomic prediction and selection (Dekkers, 2007; Duenk et al., 2021). These strategies to improve rates of genetic improvement at the commercial level for economically important traits have been implemented in some breeding programs.

### Disease resilience phenotypes

Animal health has well-known impacts on productivity and efficiency and, thereby, can have substantial negative impacts on the environmental footprint of livestock production systems (Perry et al., 2018). While empirical studies on the effects of disease on environmental impacts are limited (Kyrizakis et al., 2024), Cadero et al. (2020) estimated using simulation that pig-fatting units that are health-impaired have nearly 6% higher CO_2_ emissions at pig system level than healthy systems, as a result of greater FCR, lower growth rates, and higher mortality. However, tools to select for increased health and disease resilience are limited, in part because of the low heritability of associated traits (in part due to the typically low incidence of disease, especially in high-health nucleus herds) and the challenge of their routine recording (Dekkers, 2025). Several strategies to select for increased disease resistance or resilience have, however, been proposed, including using genetic indicator traits that can be measured on young healthy animals (Dekkers, 2025) and patterns in longitudinal data, such as daily feed intake, body weight, or activity (Harzilius et al., 2020). In addition to direct impacts on environmental footprints, improving resistance to disease will also reduce the use of antibiotics and other medical treatments that can be excreted through manure and that have an impact on environmental sustainability and human health (Pexas and Kyriazakis, 2023).

### Heat stress

Heat stress reduces productivity (Renaudeau et al., 2011; Gonzalez-Rivas et al., 2020) and increases respiration rates (Brown-Brandl et al., 1998), which is the main avenue for evaporative heat loss in pigs, since they lack sweat glands. Reduced productivity and higher respiration rates both result in a greater environmental footprint (Pexas and Kyriazakis, 2023) but can be reduced if pigs are selected to be less susceptible or more tolerant to heat stress. The importance of such selection will increase in the future with continuing climate change. Genetic differences in response or robustness to heat stress have been established (Gourdine et al., 2021), including genotype by environment interactions for grow-finish pigs (Zumbach et al., 2008; Fragomeni et al., 2016; Rauw et al., 2017; Gourdine et al., 2019) and for reproductive sows (Tiezzi et al., 2020). Strategies to select for increased heat stress tolerance include evaluating performance under thermoneutral and heat stress conditions as separate but genetically correlated traits (e.g., Dodd et al., 2022) and analysis of performance using reaction norms on temperature-humidity index data or on heat load functions thereof (Zumbach et al., 2008; Tiezzi et al., 2020). However, both these approaches have limited power when using nucleus data because of the typically greater environmental control in nucleus herds. Instead, these approaches require extensive performance data from commercial farms under diverse climate conditions.

The state of knowledge of the genetics of thermoregulation and heat stress in pigs was reviewed by Gourdine et al. (2021), which they concluded is limited for pigs. They also reviewed potential indicator traits for thermoregulation and heat stress, including physiological traits such as rectal or skin temperature and respiration rate, as well as biomarkers such as levels of metabolites and hormones that are related to thermoregulation in blood or saliva. However, little is known about the heritabilities of such indicator traits and their genetic correlations with heat tolerance and other production performance traits (Gourdine et al., 2021), nor whether measurement of these indicator traits in nucleus herds is correlated with heat tolerance under field conditions. Another category of indicator traits related to heat tolerance that was reviewed by Gourdine et al. (2021) consists of traits derived from longitudinal performance or behavior data under varying climatic conditions, such as daily feed or water intake or behavior and body weights for grow-finish pigs, and reproduction data across parities for sows (Berghof et al., 2019).

## Opportunities to enhance genetic selection to reduce the environmental impact of pork production by including novel traits related to nutrient use efficiency

The traits that are routinely recorded in breeding programs (whether on purebreds in nucleus herds or on crossbreds in the field), in particular those related to feed efficiency, are rather crude measures of the ability of a pig to use nutrients for productive purposes, rather than excreting them into the environment through urine, feces, and respiration. To address this, several traits have been suggested to provide more detailed information on an animal’s ability to utilize nutrients from the diet. These include the ability of the animal to digest or retain different components of the diet, including dry matter, organic matter, energy, crude protein, crude fat, crude fiber, non-starch polysaccharides (**NSP**), ash, nitrogen (N), and phosphorus (P). Other traits that have been suggested include serum metabolites and fecal microbiota composition, stomach acidity, muscular activity of intestinal tissue, enzyme and bile salt production, composition and activity in the small intestine, length and density of small intestinal villi, and passage rate of digesta in the total gastro-intestinal tract (Verschuren, 2021).

For their use in genetic selection programs, traits must be heritable, easily measured on a large scale on live animals (i.e., selection candidates), and not have strong unfavorable genetic correlations with other traits of interest. Thus, much focus in the use of genetics to reduce the environmental impact of pork production has been on estimation of genetic parameters of ‘novel’ traits, including heritabilities and genetic correlations with other traits, and on technology to enable cost-effective measurement on a large scale and on live animals. The primary focus of the study of these traits has been on the grow-finish phase because it is the main contributor to environmental impacts of pork production and provides a reasonable approximation for the whole system (Monteiro et al., 2016). However, genetic improvement to reduce environmental impacts of the grow-finish phase is expected to translate to some degree to parallel reductions in environmental impacts of the reproduction phase of pork production because of favorable genetic correlations, not only with regard to sow feed efficiency but also other traits. In fact, a recent study by Bouquet et al. (2022) suggested that selection of maternal lines for digestive efficiency in the grow-finish phase would result in heavier and more homogeneous piglets at birth, though from slightly smaller litter sizes but better with survival.

### CO_2_ emission

In a review of LCA studies for pork production, Philippe and Nicks (2015) noted that most LCA studies exclude the production of CO_2_ from respiration or manure, as recommended in the 2006 IPPC guidelines (Dong et al., 2006), in part because CO_2_ from respiration is offset by the CO_2_ consumption through photosynthesis of the plants that provide feed ingredients. CO_2_ exhalation in animals is related to O_2_ consumption through the respiratory quotient, which is the ratio of the volume of CO_2_ production and O_2_ consumption (Philippe and Nicks, 2015). This ratio has been estimated to be around 1.1 for growing pigs, around 1.0 for piglets, and around 0.9 for reproductive sows (Philippe and Nicks, 2015), resulting in estimated average productions of 1.7 kg CO_2_ per fattening pig per day and of 2.23 and 3.68 kg, respectively, per gestating and lactating sow per day (CIGR, 2002).

Production of CO_2_ from respiration can also be estimated from heat production, using the approximation that 1 kJ of heat produced corresponds to 24.6 L CO_2_ exhaled (da Silva and Maia, 2012). Heat production of grow-finish pigs depends on body weight and net energy intake (van Milgen et al., 2008) but is also known to differ substantially between lines of pigs (Galassi et al., 2015; Kiarie et al., 2015). For example, a partitioning of total heat production in its different components by Barea et al. (2010) indicated that fasting heat production was on average 10% lower in a line of pigs selected for low RFI (more feed efficient) at INRAE (France) than for pigs from the high RFI line. Similarly, using pigs from a similar divergent selection experiment at Iowa State University, starting at approximately 75 kg body weight, Boddicker et al. (2011) reported that pigs from the low RFI line required 7.6% less feed across a 6 wk weight stasis trial to maintain body weight and 18% less feed at the end of the trial.

Thus, although pigs produce substantial CO_2_ related to maintenance and there are known genetic differences in maintenance requirements, with animals that have lower maintenance requirements producing less CO_2_ from respiration per kilogram of pork, the impact of these genetic differences in maintenance requirements on GHG emissions are captured by their impact on feed efficiency. Including the impact differences in maintenance requirements on CO_2_ produced from respiration would result in double-counting, as they are offset by CO2 captured by the direct and indirect contributions of plants to feed.

Carbon dioxide is, however, also produced from manure in a substantial manner, depending on manure management, ranging from 4 to 40% of the production of CO_2_ from respiration, as reported in the review by Phillipe and Nicks (2015). These emissions originate from 3 processes (Phillipe and Nicks, 2015): 1) hydrolysis of urea into NH_3_ and CO_2_; 2) anaerobic fermentation of organic matter into intermediate volatile fatty acids, CH_4_, and CO_2_; and 3) aerobic degradation of organic matter. Animal factors that contribute to CO_2_ emissions from manure, therefore, include urea production (i.e., N efficiency) and digestibility of organic matter. These will be covered in a subsequent section.

### Methane emissions from enteric fermentation and manure

Most LCA studies for pigs do include CH_4_ emissions from enteric fermentation because of the greater environmental impact of CH_4_, compared to CO_2_ (Philippe and Nicks, 2015; Gislason et al., 2023). Enteric methane is the product of methanogenic Zaea known as methanogens, which are part of the digestive tract microbiota of many mammals, including pigs, and are characterized by their ability to produce methane from byproducts of bacterial anaerobe fermentations, such as H_2_, CO_2_, and formate (Guindo et al., 2020). In ruminants, their main activity is in the rumen, resulting in emissions of CH_4_ through eructation. In the pig, their main activity is in the hindgut, resulting in emissions through flatulence and feces. Misiukiewicz et al. (2021) and Yang et al. (2023) reviewed literature on the role of methanogens in the gut of pigs on methane emissions. Although the amount of CH_4_ emission from enteric fermentation is much larger in ruminants, Vermorel et al. (2008) and Dämmgen (2012) estimated daily enteric CH_4_ emissions of 0.8 and 0.9 g, respectively, per head for weaned piglets (up to 20 kg), of 2.4 and 2.5 g for fattening pigs (from 20 kg), and of 8.2 and 6.1 g per head for reproductive sows, under French and German production conditions, respectively.

In addition to age, the amount of methane produced by pigs is mostly related to how much digestible residue enters the hindgut, which is defined as the difference between digested organic matter and digested protein, fat, starch, and sugar, along with the fermentation capacity of the hindgut (Philippe and Nicks, 2015). The latter is in part determined by the genetics of the pig, possibly through its influence on the gut microbiome, in particular methanogens. Luo et al. (2012) showed that Landrace pigs of various ages (40 d to 1 yr) had a greater abundance and diversity of methanogens than same age pigs from the Erhualian breed, which is a local Chinese breed with greater fatness. *Methanobrevibacter*-like sequences were dominant in the Erhualian breed (96.6% vs. 56.5% for Landrace), with most of the remaining methanogen-related sequences comprising *Methanosphaera*-like sequences (3.4 and 43.5%, respectively). In rabbits, divergent selection for intra-muscular fat has been shown to alter the composition of the gut microbiota, with the low intra-muscular fat line having a greater abundance of *Methanobrevibacter* (Martínez-Álvaro et al., 2023). In humans, *Methanobrevibacter smithii* is the predominant methanogen in the gut and its abundance has been found to be substantially heritable (20%) in a study of UK twins (Goodrich et al., 2016). In addition, abundance of methanogens in the human gut has been found to be correlated with abundance of the bacterial family *Christensenella*, whose abundance is the most heritable (0.42) of all archaea in the human gut (Goodrich et al., 2016). In a meta-analysis, Ruaud et al. (2020) showed that the bacterial family *Christensenellaceae* and the archaeal family *Methanobacteriaceae* co-occur and are enriched in individuals with a lean, compared to an obese, body mass index. The latter agrees with findings in pigs regarding the abundance of methanogens in the feces of lean vs. fat breeds (Luo et al., 2012). Using co-culture, Ruaud et al. (2020) also showed that *Christensenella* supports the metabolism of *M. smithii* via H_2_ production in humans much better than other bacterial species. This use of H_2_ has been hypothesized to result in increased carbon loss, thereby reducing energy uptake. The substantial correlation and heritabilities of the abundances of these 2 species in the human gut microbiome, combined with their close physical and metabolic interactions, suggest potential heritable roles in enteric methane production and could explain their associations with leanness in humans (Schwiertz et al., 2010; Million et al., 2012) and pigs (Luo et al., 2012). Methanogens have also been linked to immune response under various conditions of health and disease in humans (Borrel et al., 2020; Thomas et al., 2022).

After excretion by the animal, under the right conditions, acetate, CO_2_, and H_2_ are converted into methane by methanogenic bacteria in manure (Philippe and Nicks, 2015). Methane emissions from manure are proportional to the amount of volatile solids or organic matter in the manure by a factor that depends on the manure management practice (IPCC, 2006). On average, for manure management systems in temperate Western Europe, Philippe and Nicks (2015) reported the production of 33 g CH_4_ from manure per head per day, based on IPCC (2006) estimates. This is over 10 times as large as enteric methane production. Methane emissions from manure can be reduced by increasing organic matter digestibility by the pig which, given the potential magnitude of these emissions, can have substantial effects. The genetic basis of organic matter digestibility is addressed in the next section.

### Nitrogen (or protein) and phosphorus efficiency

Several reviews have identified genetic selection for increased N and P utilization efficiencies as promising approaches to achieve a reduction in nutrient supply and nutrient voiding (Merks et al., 2012; Neeteson-van Nieuwenhoven et al., 2013; Millet et al., 2018). Nitrogen and phosphorus efficiency are defined as the amount of N or P retained in the body as a ratio of the amount of N or P consumed. Thus, their measurement on individual pigs requires measurement of N (or protein) and P intake through feed and measurement of the amount that is retained in the body. The latter can be determined by measuring the concentration in the body, along with body weight, or by measuring the amount that is excreted through urine and feces. Shirali et al. (2012) estimated that approximately 51% of N intake is excreted in urine, which is mainly from protein metabolism, underutilized amino acids, and non-protein N. Nitrogen excreted from feces accounts for on average 17% of N intake and is driven by undigested protein fractions and endogenous tissue losses (Dourmad et al., 1999). Using a standard wheat–barley–soybean meal diet that was marginal for lysine fed to 40 to 70 kg pigs, Berghaus et al. (2023) showed that two-thirds of total N excreted by grow-finish pigs is excreted in the urine and, of that, 80% is present in the form of urea. For P, Dourmad et al. (1999) estimated that only approximately 30% of P is retained in a grower-finisher pig on a cereal–soybean meal-based diet, with the remainder excreted through the feces and urine. The percentage retained is, however, substantially increased when phytase is included in the diet.

#### Feed intake:

Recording individual feed intake on group-housed grow-finish pigs is now routine in nucleus breeding programs, with the use of single-space electronic feeders. Combined with estimates of diet composition, from chemical analyses or near-infrared spectroscopy (**NIRS**), this allows intake of different diet components during a specific time period to be estimated on individual animals. It should be noted that these estimates are affected by feed wastage, which depends on feeder design, among others. In addition, feed intake and feed intake behavior obtained from such single-space feeders, often with protective side walls to reduce simultaneous feeding of more than one pig, can be different from what would be observed in commercial multi-space feeders (Casey and Dekkers, 2003).

#### Retention:

Several methods are available to estimate N or P retention in grow-finish pigs, using invasive and non-invasive methods. Chemical analysis of the whole carcass is the gold standard but this is time consuming, expensive, and destructive. Approximate methods to estimate N retention indirectly are based on lean body mass and the standard N content of lean mass. Estimates of lean mass can be obtained based on i) weight of primal cuts, ii) body weight and backfat thickness (carcass or ultrasound), iii) empty body water content based on deuterium dilution (Landgraf et al., 2006), iv) empirical analysis of feed intake and growth curves (Verschuren et al., 2021), v) dual-energy X-ray absorptiometry (DXA) scans, and vi) magnetic resonance imaging (**MRI**) (Delgado-Pando et al., 2021). Non-evasive technologies to measure body composition on the live animal were recently reviewed by Scholz et al. (2021). Most of these indirect methods rely on assumptions that require prediction equations that are specific to a breed and/or sex (Scholz et al., 2015), for example, because of the use of composition estimates obtained at specific points on the body (e.g., backfat). Kasper et al. (2021) showed that accurate estimates of carcass N content (R^2^ > 0.98) can be obtained using whole body DXA estimates of lean mass. Estimates of carcass content obtained from DXA estimates of lean mass and bone mineral content were less accurate (*R*^2^ approximately 0.87) because of the lower accuracy of estimating bone mineral content (Kasper et al., 2021). DXA scans utilize information from the entire body and their application may, therefore, be less sensitive to breed and sex differences. DXA scans can also be obtained on live animals and this has been implemented in several nucleus breeding programs (Scholz et al., 2021).

#### Excretion:

Dietary nutrients are excreted in both urine and feces. Using grab sampling, collecting fecal samples on individual pigs on a large scale is possible, but collection of urine samples is challenging. Fecal samples can be used to estimate excretion of diet components, including N and P, by measuring their concentration in the sample compared to the concentration of an inert marker that was added to the feed at a known concentration, or by using acid-insoluble ash as an inert marker (Sales and Janssens, 2003). This allows apparent total or fecal digestibility of diet components to be estimated on an individual animal basis. Expensive laboratory analyses and the use of an inert marker can be avoided by using NIRS analysis of a sample to predict the chemical composition of feed and feces (Bastianelli et al., 2015). Labussière et al. (2019) developed equations to predict nutrient digestibility of grower-finisher pigs based on NIRS of fecal grab samples, with prediction accuracies of 0.93 for dry matter, 0.94 for organic matter, 0.94 for energy, 0.95 for crude protein, and 0.82 for crude fat digestibility. Déru et al. (2021) used the NIRS equations developed by Labussière et al. (2019) to predict nutrient digestibilities for genetic parameter estimation. Paternostre et al. (2021) further showed that combined analysis of NIRS spectra from feed and fecal samples resulted in more accurate estimates of nutrient digestibility than using separate analyses of feed and fecal NIRS spectra.

Although excretion of N through urine is difficult to quantify directly, blood urea N (BUN) concentration has been shown to provide a relatively easy-to-measure indicator trait for N excretion in urine (Kohn et al., 2005). The correlation between BUN and N excretion in urine is high when comparing diets with different protein levels (0.84, Zervas and Zijlstra, 2002) but only moderately high when comparing animals that are fed the same diet (0.5, Berghaus et al., 2023). The latter is relevant to the use of BUN in urine as an indicator trait for genetic selection.

### Estimates of heritability of digestibility and retention of diet components

Using retention studies based on body weight and composition, Saintilan et al. (2013) estimated significant heritabilities for N retention (0.36 to 0.43) and for P retention (0.30 to 0.41) in grow-finish pigs. Using chemical analyses of the carcass, Kasper et al. (2020) estimated heritabilities of 0.41 for N efficiency, while P efficiency was not heritable. Ewaoluwagbemiga et al. (2023) estimated heritabilities of protein efficiency and P efficiency of 0.54 and 0.27, respectively, based on estimates from carcass DXA scans. The genetic correlation between protein and P efficiency was estimated to be positive, at 0.61. Schmid et al. (2024) estimated heritability of N utilization efficiency, estimated using prediction equations derived from N balance data and blood metabolites on a subset of the 508 Landrace × Pietrain pigs evaluated, to be 0.29 and 0.16 at, respectively, 13 and 16 wk of age. The estimate of the genetic correlation of N efficiency between these 2 ages was, however, less than 1 (0.73±0.21).

Using empirical estimates based on growth and feed intake curves, Verschuren et al. (2021) estimated heritabilities of N efficiency to range from 0.21 to 0.27 across the starter, grower, and finisher phases. The genetic correlation of N efficiency was high (0.92) between the starter and grower phases, but low between these 2 phases and the finisher phase (0.13 and 0.47, respectively).

Estimates of heritability of apparent total track fecal digestibility of N using NIRS and standard diets have been estimated to be moderate, 0.27 by Déru et al (2021) and 0.20 by Hov Martinsen et al. (2023). Déru et al (2021) estimated heritability and genetic variance of N digestibility to be substantially greater when pigs were fed a high-fiber (0.56) vs. a conventional diet (0.27), which suggests that genetic evaluation for N digestibility should be conducted on high-fiber diets.

Estimates of the heritability of digestible residue, relevant to enteric methane production, are not available. However, with regard to the level of methane production from manure, several studies have estimated the heritability of fecal digestibility of organic matter to be moderate, ranging from 0.20 (Hov Martinsen et al., 2023) to 0.27 (Déru et al., 2021).

BUN, as an indicator for urine N excretion, has been shown to have moderate heritability; 0.16 to 0.35 by Klindt et al. (2006) and 0.42 and 0.46 at, respectively, 13 and 16 wk of age by Schmid et al. (2024). The latter, however, found the genetic correlation of BUN at these 2 age to be close to 1 and also to be genetically correlated with N use efficiency at those ages (−0.52 to −0.63).

These heritability estimates demonstrate that it is possible to select for improved digestibility and efficiency of the use of nutrients to minimize environmental impacts of pork production through their excreta. Further research is, however, needed to establish the best methods and conditions (i.e., age, diet, feeding strategy) to measure these traits.

### Genetic correlations of N and P efficiency with overall feed efficiency

The need for recording the efficiency with which a pig uses individual diet components such as N, P, and organic matter, in order to enhance selection to reduce environmental impact through excreta depends, among others, on the genetic correlation of these measures with traits that are already routinely recorded in breeding programs, in particular with overall feed efficiency or FCR. If these genetic correlations are high, separate recording of the efficiency of the use of individual diet components is not necessary. Saintilan et al. (2013) estimated genetic correlations of N and P efficiency traits with FCR to be very close to −1 for all investigated breeds, estimating retention of N and P from body weight and lean mass content of the carcass based on carcass cut weights. Using N and P retention estimated from carcass DXA scans under a protein-restricted diet, Ewaoluwagbemiga et al. (2023) estimated the genetic correlation of N efficiency with FCR to be moderately negative (−0.55 ± 0.14). Using fecal excretion estimates obtained from fecal grab samples obtained just prior to entry into the finishing phase (approximately 16 wk of age), Déru et al. (2021) estimated moderate negative genetic correlations of fecal N digestibility with both FCR (−0.27 and −0.34 for a conventional and high-fiber diet) and ADG (−0.30 and −0.33) but higher negative genetic correlations with ADFI (−0.59 and −0.41) and especially with RFI on a conventional diet (−0.83 and −0.50). Martinsen et al. (2023) estimated significant negative genetic correlations of fecal N digestibility with ADFI (−0.54) and backfat (−0.31) but estimates were not significantly different from zero for ADG (+0.11) and loin depth (−0.11).

The above studies considered efficiency traits across the grow-finish phase. However, Verschuren et al. (2021), estimating N retention based on growth and feed intake curves using the INRAPorc growth model (van Milgen et al., 2008), found estimates of genetic correlations between N efficiency and FCR to change during the growth period, with moderately negative estimates in the starter phase (−0.47) that became more negative thereafter, up to −0.90 in the finisher phase. The latter suggests that most genetic variation in N efficiency in the finisher phase is indeed explained by FCR. These correlation estimates may, however, have been affected by the method used to estimate N efficiency, which was based on empirical relationships between growth and feed intake curves, potentially increasing the magnitude of genetic correlation estimates with FCR.

Soleimani and Gilbert (2020) used data from the fifth generation of a divergent selection experiment for RFI in Yorkshire pigs to evaluate the effect of such selection on multiple environmental impact categories. The INRAPorc nutritional growth model (van Milgen et al. 2008) was used to model growth performance and composition for each grow-finish pig based on its recorded phenotypes (regular body weights, feed intake during fattening, and ultrasound backfat prior to slaughter), resulting in estimated phenotypes for protein deposition during fattening and protein and lipid body weights at slaughter. The resulting phenotypes were integrated with an individual-based LCA model to estimate the environmental impact of each pig for each environmental impact category. Results showed that pigs from the low RFI line (more feed efficient) had on average 7% lower environmental impacts per kilogram of live slaughter weight than pigs from the high RFI line. Interestingly, the difference in FCR between the 2 lines was also 7%. In addition, FCR had a phenotypic correlation that was greater than 0.96 with each environmental impact category across animals in both lines, indicating that FCR captures most of the phenotypic variation in environmental impacts. The phenotypic correlations of RFI with environmental impact categories were, however, lower, ranging from 0.71 to 0.75 in the low RFI line and from 0.51 to 0.55 in the high RFI line. The lower correlations for RFI vs. FCR are because, in contrast to FCR, RFI does not capture differences in growth rate, which is negatively correlated with environmental impacts (faster-growing pigs have a lower environmental impact). Interestingly, the ratio of body protein to body lipid at slaughter, as estimated using the INRAPorc growth model, had high negative phenotypic correlations with environmental impacts, of −0.68 for each impact category for the low RFI line and ranging from −0.74 to −0.83 for the high RFI line. The high correlation between FCR and environmental impacts of individual pigs was confirmed in a simulation study based on the INRAPorc model by Monteiro et al. (2021). In fact, this study showed that N efficiency and N excretion, which are responsible for most of the direct environmental impacts of growing pigs, were both highly correlated with FCR (<−0.95 and >+0.95, respectively), but that FCR was more highly correlated with each environmental impact category (> 0.99) than N efficiency (ranging from −0.96 to −0.99) or N excretion (0.96 to 0.97). Interestingly, these correlations were little affected by using 2-phase vs. precision feeding, although the latter did reduce the average magnitude of the environmental impacts.

Using complete N balance studies, van der Peet-Schwering et al. (2021) showed that grow-finish pigs with a high EBV for protein deposition have a higher apparent N efficiency (55.8%) and N retention than pigs with a low EBV (52.7%), at least when fed a diet with adequate protein levels. However, when fed a protein-restricted (70%) diet, these differences disappeared. Thus, in precision feeding to optimize protein and amino acid efficiency in pigs, differences in protein deposition must be considered as a factor.

Related to excretion of N through urine, Schmid et al. (2022) estimated BUN to be genetically uncorrelated with FCR (−0.06) but positively correlated with ADFI (0.44) and ADG (0.33).

The substantial heritabilities show that selection for increased N efficiency is possible, while less than unit genetic correlations with FCR (favorable), RFI (favorable), ADFI (favorable), and ADG (unfavorable) indicate that N efficiency captures genetic variation that is not fully captured by routinely recorded traits, although estimates are not consistent across studies. If genetic correlations of N efficiency with traits that are under selection (e.g., FCR, RFI) are indeed as high as suggested in some studies, opportunities to further enhance genetic selection for N efficiency may be limited. However, in poultry, based on estimates of genetic parameters, De Verdal et al. (2011) showed that direct selection for N or P excretion traits has the potential to be much more effective in reducing excretion than selection on FCR or RFI. A complete and accurate set of genetic parameters among all traits is required to evaluate this for grow-finish pigs. In addition, genetic correlations between efficiency and excretion traits appear to change with developmental stage of grow-finish pigs, which requires further investigation to determine the optimal time(s) to measure and select for N efficiency. However, since the environmental impact of pork production is greatest during the finisher period (high feed intake and manure production), the major focus should be on that phase.

### Water use

In many areas of the world, water is a scarce resource that can limit food security (Rijsberman, 2006). Agricultural production accounts for more than 70% of global water use, of which nearly one-third is associated with farm animal production (Mekonnen and Hoekstra, 2012). Up to 98% of the latter is associated with feed production (Ibidhi and Ben Salem, 2020). In a review of the application of LCA to pig and poultry production, Andretta et al. (2021) noted that many studies did not include water use as an impact category or they did not consider water consumption. Ibidhi and Ben Salem (2020) identified 3 options to reduce the water footprint of livestock production: (1) reducing the water footprint of feed production, (2) increasing the efficiency of the use of feed resources, and (3) drinking water and animal management. The latter includes the use of animals that use water more efficiently.

Knowledge of the genetics of water use by pigs is, unfortunately, limited. Hollifield et al. (2024) estimated genetic parameters of time at the drinker and at the feeder in purebred grow-finish pigs housed in groups based on digital phenotyping. They estimated drinking time to have substantial heritability (0.38) but lower than that of feeding time (0.57). Feeding and drinking time had moderately high genetic (0.38) and phenotypic (0.17) correlations. Cheng et al. (2021) estimated genetic parameters of water use in the finisher under a polymicrobial natural disease challenge. The average water disappearance was 5.1 L/d, with a standard deviation of 2.4 L/d. Traits related to drinking were found to have moderately high heritabilities (0.34, 0.44, and 0.54 for average water disappearance, time at the drinker, and number of visits to the drinker per day). Estimates of genetic and phenotypic correlations between water disappearance and time at the drinker were moderately high, at 0.68 and 0.60, respectively, suggesting that time at the drinker may not be a very accurate indicator trait for water use. Similar to Hollifield et al. (2024), Cheng et al. (2021) estimated the genetic and phenotypic correlations between drinking and feed intake traits to be fairly low, i.e., 0.36 and 0.39, respectively. Water disappearance also had a much lower genetic correlation with growth rate (0.29) than feed intake did (0.84). The sizeable heritabilities and standard deviations and reasonably low correlations with feed intake and growth rate indicate that opportunities exist to select for reduced water use without severe impacts on production traits. Further research is, however, needed to investigate these genetic parameters without the presence of major disease. In addition, assuming that a portion of water disappearance is the result of playing with the drinker, the impact of alternate designs for the drinker needs to be evaluated. The recording of phenotypes related to drinking is now facilitated by the use of cameras. Using video systems and machine learning, Zhu et al. (2017) were able to identify drinking behavior of individual pigs in group housing, while Chen et al. (2020) developed methods to differentiate drinking and play behavior of pigs.

## Interaction of genetics and nutrition

Pigs are raised under a wide range of conditions and are fed a wide range of diets. With increased emphasis on formulation of diets that reduce environmental impacts, the variety of diets that pigs will be fed is expected to increase, with a move towards increased use of byproducts and/or ingredients with lower energy contents. This raises the question of the presence of genotype by diet interactions, i.e., whether the ranking of lines and of animals within lines based on performance (or environmental impact) is expected to be the same when they are fed different diets. The presence of such interactions has substantial implications for the diets that should be fed to animals that generate the phenotypes that inform selection decisions in breeding programs through genetic evaluation.

As described previously, many studies have shown that the genetic correlation of purebred performance in the nucleus with crossbred performance in the field can be substantially less than one. There are 3 factors that can contribute to these genetic correlations being less than one, as described by Wientjes and Calus (2017): i) genotype by genotype interactions (i.e., purebred vs. crossbred genetics, resulting in different dominance and epistatic effects if non-additive genetic effects are important), ii) genotype by environment interactions (GxE, i.e., resulting from differences in the expression of the same genetics in nucleus vs. commercial environments), and iii) differences in measurement of the trait at the purebred nucleus and commercial crossbred levels. Based on limited published literature available, Wientjes and Calus (2017) also surmised that genotype by genotype interactions (non-additive genetic effects) appeared to be the main contributor to the low genetic correlations. However, differences in environments in these studies may not have been as extreme as can be expected in practice and, thus, GxE is likely also a substantial contributor. Presence of GxE can be the result of differences in diets fed at the nucleus vs. commercial levels, as well as differences in health status and management, among others.

Few studies are available that directly quantify genotype by diet interactions. Wallenbeck et al. (2009) observed some re-ranking of boars based on growth and backfat thickness in progeny evaluated under conventional vs. organic production systems. Similarly, comparing the performance of commercial and local breeds under conventional high-input and organic production systems with organic diets based on farm-grown feedstuffs, Brandt et al. (2010) observed GxE for growth performance and carcass quality, although there was limited re-ranking of breeds. Godinho et al. (2018) analyzed GxE for performance of pigs on a corn–soybean diet vs. a wheat–barley-coproducts diet (i.e., a low-input diet from an environmental impact standpoint), but with similar net energy and crude protein contents and a similar net energy/digestible lysine ratio during different phases of grow-finish. They found that diet differences did not cause GxE for the main performance traits of growth rate, protein deposition, feed intake, and FCR during any phase. However, substantial GxE was observed for lipid deposition and for residual feed and energy intake as measures of feed efficiency. The presence of such GxE suggests that, if commercial diets in the future are formulated to reduce environmental impacts, genetic selection in nucleus herds should be on feed efficiency under a low-input diet.

Mauch et al. (2018) observed that differences in performance and feed efficiency between 2 lines of Yorkshire pigs that were divergently selected for feed efficiency based on RFI under a higher-energy, lower-fiber diet were less when pigs from these lines were instead fed a low-energy, high-fiber diet. However, genetic correlations of performance trait between the 2 diets tended to be high, ranging from 0.87 for RFI to 0.99 for loin muscle area, providing limited evidence of GxE.

Although evidence for the presence of substantial genotype by diet interactions is limited and more research on the impact on a wider range of diets is needed, these findings do suggest that selection criteria and conditions under which phenotypes for selection are obtained may need to be changed in order to achieve optimal genetic improvement under more sustainable low-input diets. As reviewed by Phocas et al. (2016), genetic improvement to increase sustainability requires selection criteria to be targeted to the range of environments that the improved genetics is expected to be expressed under, as well as for genetic improvement that is robust across environments, including across a range of diets. This requires strategies for improvement of sustainability of pig production through genetics and nutrition to be integrated (Phocas et al., 2016), as also exemplified by the work of Solemeini et al. (2021) discussed above.

## Integrating genetics and nutrition

Opportunities to reduce environmental impacts by tailoring diets to genetics were investigated by Soleimani and Gilbert (2021) and Soleimani et al. (2021), with application to quantifying differences in environmental impact between the INRAE lines of Yorkshire pigs that were divergently selected for 5 generations for high vs. low RFI on a conventional diet. Under the conventional diet, the low and high RFI lines differed (high RFI minus low RFI) in RFI by 0.071 kg/d, in ADG by 0.03 kg/d (3.7%), in ADFI by 0.18 kg/d (5.1%), and in FCR by 0.13 kg/kg (5.2%). Differences in environmental impacts between the 2 lines under the conventional diet ranged from 6.0 to 8.0% in favor of the low RFI line (Soleimani and Gilbert, 2020). Thus, differences in environmental impacts between the lines were greater than differences in performance. Soleimani and Gilbert (2021) expanded on this work by developing environmentally sustainable diets, separately for each line; using the INRAPorc nutritional growth model (van Milgen et al., 2008). These line-specific diets were developed as the diet that, given available ingredients, minimized environmental impacts (the sum of impacts on GWP, TAP, FEP, and ALU), while meeting the nutritional requirement of the line in terms of digestible crude protein, digestible lysine, digestible threonine, digestible tryptophan, and digestible methionine, all expressed relative to the diet’s net energy content, which was assumed to be the driver for ad libitum feed intake. Performance and excretion of individual pigs from the 2 lines under these alternate diets were then simulated based on the INRAPorc nutritional growth model (van Milgen et al., 2008), which were fed into the LCA developed by Soleimani and Gilbert (2020), separately for each pig, and averaged to quantify the environmental impacts of each line under its tailored diet. Soleimani et al. (2021) further expanded the work of Soleimani and Gilbert (2021) by adding economics to diet formulation options (least-cost diets) and to the overall system objective. They showed that the low RFI line generated substantially greater profit than the high RFI line under the conventional diet (23.4% difference) and also under LC diets (18.5%) and under least environmental impact (**LEI**) diets (21.8%) that were tailored to meet the nutrient requirements of each line. However, under diets that were tailored for each line to minimize a combination of cost and environmental impacts, the difference in profit was only 7.6%. The low RFI also had a lower overall environmental impact (sum of the 4 impacts) for all diets, with differences between the 2 lines at 7.2% and 8.1% for the conventional and LC diets and almost half of that for the LEI diet (4.9%) and the diet that jointly minimized cost and environmental impacts (4.3%). It should be noted that these comparisons were based on the average of outcomes of nutritional, bio-economic, and LCA models that were applied to the observed phenotypes of individual animals and are, therefore, subject to random errors. Nevertheless, the results demonstrated that optimization of diets and nutrition to minimize cost and environmental impacts tailored to genetics of the pig can substantially reduce environmental impacts while limiting reductions in profit compared to LC rations that are tailored to genetics. In addition, optimization of diets to minimize cost and environmental impacts tailored to genetics substantially reduced differences in profit and environmental impacts between pigs that differ in feed efficiency. This could have substantial implications for the diets that pigs should be selected under. In the study by Soleimani et al. (2021), considering ingredients available in Western Europe, LC and LEI diets primarily differed in the proportion of triticale (lower for LEI compared to LC diets and for the high compared to the low RFI line), of barley and wheat (higher for LEI diets), of corn (lowest for the LC diet for the low RFI line, highest for the LC diet for the high RFI line, and intermediate for the LEI diet for both lines), of peas (high for the LEI diet for the high RFI line, absent in the LEI diet for the low RFI line, and intermediate for the LC diets), of rapeseed meal (higher for LEI diets), of sunflower meal (approximately 0 for LEI diets), of soybean meal (only sizeable in the LC diet for the high RFI line).

Rather than developing line-specific diets, as explored by Soleimani et al. (2021), it is also possible to develop animal specific diets, as has been proposed and developed by several (Pomar et al., 2009; Monteiro et al., 2016; Remus et al., 2019). By tailoring energy and amino acid supply to their requirements by individual pigs on a day-to-day basis, these precision feeding approaches have been shown to have the potential to substantially reduce environmental impacts, while improving performance and profitability. While current approaches are based on estimation of future nutritional requirements of the animal based on longitudinal phenotypic data collected on the animal up to that point in time (Hauschild et al., 2012; Brossard et al., 2017; 2019), these estimates could be enhanced by using genetic estimates of different aspects of performance over time for the line and/or for each animal.

To this end, methods that integrate nutritional and genetic evaluation models, such as those developed by Yu et al. (2022) for grow-finish pigs, can be beneficial. In their approach, a multi-trait genomic prediction model for (unobserved) physiological traits or latent variables is linked to observed phenotypes (longitudinal body weights and feed intake and body composition at one of more ages) through a mechanistic nutritional growth model. By EBV for the underlying physiological traits that drive growth and feed intake, these models are hypothesized to enable prediction of performance under different diets. This is in contrast to current genetic evaluation models, which predict breeding values for growth rate and feed intake for the diet that the phenotypes were collected under. This concept of integrating nutritional growth models into genomic prediction models was inspired by approaches that have been developed for prediction and genomic evaluation of genetic lines of corn for yield under different environmental conditions (e.g., drought) by integrating crop growth models into methods for genomic prediction (Technow et al., 2015). In the crop growth models, underlying physiological traits or characteristics for which breeding values are estimated and that selection is proposed to be on include the ability of the plant canopy to capture and use sunlight, such as total leaf number, area of largest leaf, solar radiation use efficiency, as well as thermal units to physiological maturity (Technow et al., 2015; Cooper et al., 2016). Messina et al. (2018) demonstrated that the integration of suitably parameterized crop growth models into methods for genomic prediction can capture genotype by environment by management interactions and predict yield under a range of environments and management situations.

Applied to longitudinal data on body weight and feed intake of grow-finish pigs, Yu et al. (2022) integrated components of a simplified version of the INRAPorc pig growth model of van Milgen et al. (2008) with underlying genetic traits that included 3 variables for the Gompertz curve for body weight (related to mature weight and shape of the growth curve) and 2 variables that model feed intake as a function of body weight. Recently, these models were extended by modeling body composition, including a Gompertz curve for protein deposition and lipid deposition as a function of energy intake and biological parameters (Yu et al., 2023). Further development of these models will enable integration of the use of specific nutrients, including essential amino acids, as, e.g., in Soleimani et al. (2021).

Models that integrate nutrition and genetics have applications at both the commercial production level and in genetic improvement programs, aiming to select lines that are tailored to specific environments, diets, or nutritional strategies. Selecting animals for growth when fed diets that are tailored to meet the specific nutritional requirements of the line, or even the individual pig, is expected to put selection pressure on improvement of efficiency of the use of specific diet components. If genotype by diet and by nutritional strategy effects are large, this could lead to the need to develop different lines tailored to different diets and nutritional strategies. However, as suggested by the work of Solemeini et al. (2021), precision feeding could also reduce interactions between genetics and diet, which would enable genetic improvement of a single set of parental lines (typically 2 maternal, e.g., Landrace and Yorkshire, and one terminal line, e.g., Duroc) to effectively translate into genetic improvement across a range of diets and nutritional strategies at the commercial level. This can be enhanced by selecting these parental lines on traits that are robust to differences in diets and environments, which is the aim of methods that integrate nutritional and genetic models of performance. The effectiveness of this approach does, however, depend on the accuracy of the nutritional models that are employed to determine the nutrient requirements of each line or animal, as well as the accuracy with which these requirements can be characterized at the genetic level.

If genotype by diet by feeding (**GxDxF**) interactions remain important, despite the above efforts, strategies to deal with them in a genetic improvement program can be substantially more effective if a clear understanding of the nutritional factors that generate these interactions is developed. For example, if GxDxF interactions are a function of the fiber content of the diet, reaction norm models can be used to compute EBV for performance as a function of fiber content. This allows performance of a line or cross to be predicted for the full range of diets with different fiber contents. Multiple-trait reaction norm models and approaches described by Dekkers (2021) can then be used to determine the optimal diet to obtain phenotypes for genetic evaluation and the optimal selection strategy to optimize performance of pigs under the range of diets used in the industry. Similar approaches can be used when relationships between traits and diet factors are nonlinear, as in Yu et al. (2022).

## Genetic modification and gene editing

Advances in genomic technologies have opened opportunities to introduce genes from other species (transgenics) and, more recently to change the genetic make-up of an animal by gene editing. See Ledesma and Van Eenennaam (2024) and Whitworth et al. (2022) for a recent review of these technologies and their current and potential future applications in, respectively, agricultural animals and pigs specifically. One application of these technologies that is directly related to the environmental impact of pork production is to provide solutions for anti-nutritional factors that many feedstuffs contain and that result in reduced availability of nutrients to the pig. For example, pigs have poor physiological ability to hydrolyze plant phytates, which accounts for up to 80% of P in common cereal grains, oil seed meals, and byproducts (Ravindran et al., 1994). Golovan et al. (2001) and subsequently Forsberg et al. (2013) reported the creation of a transgenic line of pigs that secreted bacterial phytases in their saliva. Meidinger et al. (2013) showed that transgenic barrows from this line that were fed a diet without supplemental P retained 25 to 40%, 77 to 91%, and 27 to 56% more P than non-transgenic barrows during the weaning, growing, and finishing phases, respectively.

Pigs are also poorly able to digest NSP because of a lack of endogenous NSP-degrading enzymes (NSPases) (Hooda et al., 2010), although NSPs are partially degraded by the natural microbial community in the pig’s intestinal tract. Zhang et al. (2018) created transgenic pigs that harbor a single-copy of a quad-cistronic transgene and that simultaneously express 3 microbial NSPases and phytase in the salivary glands. Fecal N and P outputs in the transgenic pigs were reduced by 23.2 to 45.8%, growth rate improved by 23.0 (gilts) and 24.4% (boars), and FCR was improved by 11.5 to 14.5%. Guan et al. (2017) produced a transgenic pig with salivary specific expression of β-glucanase to reduce the negative effects of β-glucan on nutrient absorption and growth. Similar strategies are being explored to introduce pectinases into the pig genome in order to reduce the anti-nutritional impact of the plant cell wall component pectin (Mo et al., 2019). These findings indicate that the transgenic or gene-edited pigs are promising resources for improving feed efficiency and reducing environmental impacts, as an alternative to dietary interventions such as supplementation of phytase and NSP-degrading enzymes (De Lange and Swanson, 2006, p. 553). An advantage of addressing these solutions using genetics is that no further intervention is needed after the genetic change has been introduced into a line. Such genetic interventions are, however, subject to regulatory and societal approval.

## Conclusions and research needs

By improving feed and reproductive efficiency, in particular, genetic improvement has substantially reduced the environmental impact of pork production, although future calculations should account for the possible use of manure as a source of fertilizer. Other than further accelerating current rates of genetic improvement for traits of interest, which primarily aim to reduce CoP, limited opportunities appear to exist to further accelerate reductions in environmental impacts within the context of current phenotype recording and genetic improvement programs. These assessments are, however, primarily based on LCA calculations, which greatly depend on diet formulation and on the factors that control voluntary feed intake, which emphasizes the need for additional research into the genetic control of feed intake and its interactions with diet formulation. Opportunities do exist to tailor phenotype recording programs to commercial rather than nucleus environments, which will accelerate rates of genetic improvement in the field. In addition, opportunities exist to improve selection for resilience to disease and heat stress, which are important contributors to the environmental footprint of pork production.

Additional opportunities exist to directly select to reduce environmental impacts of pork production, including enteric methane production, methane production from manure, and excretion of minerals (in particular N, P) and compounds (e.g., organic matter) by pigs. Direct and indirect methods to measure these traits are available but require further development and optimization. Further investigation into the optimal diets, feeding strategies, and ages that should be used to measure the efficiency of the utilization of different diet components, specifically N and P, is needed, such that the resulting measures have high heritability and high genetic correlations with utilization efficiency across the grow-finish period under commercial conditions and different diets. Genetic correlations of N and P efficiency with overall feed efficiency and other routinely measured traits are inconsistent but are required to determine the added value of measuring these traits in breeding programs and to formulate optimal phenotype recording programs.

Opportunities also exist to reduce the environmental impact of pork production by tailoring diets to the nutritional requirements of a line, a cross, or even an individual. However, much additional research is needed on the impact of such precision feeding strategies on genetic differences between lines and individuals, using diets that take economic and environmental impacts into account. Research is also needed to optimize phenotype recording strategies in genetic improvement programs that maximize or optimize genetic improvement at the commercial level under such feeding strategies and diets. This requires further development and integration of nutritional and genetic models. Recording and genetics of water use is another aspect with impacts on environmental footprints that requires further investigation.

Opportunities also exist to use transgenics or gene editing to provide solutions for anti-nutritional factors that many feedstuffs contain and that result in reduced availability of nutrients to the pig.

While the grow-finish phase of pork production offers the greatest opportunities to reduce environmental impacts by genetic improvement, the impact of such selection on the reproductive phase of pork production also needs to be evaluated. As well, specific opportunities to further reduce the impact of the reproductive phase on the environment must be investigated.

## Abbreviations

ADFI: average daily feed intake
ADG: average daily gain
ALU: agricultural land use
BF: backfat
BUN: blood urea nitrogen
CoP: cost of production
DXA: dual-energy X-ray absorptiometry
EBV: estimated breeding value
FCR: feed conversion ratio
FEP: freshwater eutrophication potential
FRS: fossil resource scarcity
GHG: greenhouse gas
GWP: global warming potential
GxDxF: genotype by diet by feeding interactions
GxE: genotype by environment interaction
LCA: life cycle assessment
MRI: magnetic resonance imaging; N, nitrogen
NIRS: near-infrared spectroscopy
NSP: non-starch polysaccharides
P: phosphorus
RFI: residual feed intake
TAP: terrestrial acidification potential
